# Cross-Condition Fault Diagnosis of Rolling Bearings Based on Time–Frequency Ridge Extraction and Uncertainty-Guided Distribution-Regularised Convolutional Wasserstein Autoencoder

**DOI:** 10.3390/s26175666

**Published:** 2026-09-06

**Authors:** Junshen Zhang, Jixing Yang, Xinfa Shi, Qing Zhang

**Affiliations:** 1 School of Mechanical Engineering, Xi′an Jiaotong University, Xi′an 710049, China; 3120101238@stu.xjtu.edu.cn (J.Z.); jixingyang@stu.xjtu.edu.cn (J.Y.); 2 Guangzhou Mechanical Engineering Research Institute Co., Ltd., Guangzhou 510700, China; 3 School of Instrument Science and Technology, Xi′an Jiaotong University, Xi′an 710049, China; 4 State Key Laboratory for Manufacturing Systems Engineering, Xi’an Jiaotong University, Xi’an 710049, China

**Keywords:** rotating machinery, time–frequency ridge extraction, cross-condition diagnosis, maximum mean discrepancy, uncertainty weighting

## Abstract

**Highlights:**

**What are the main findings?**

- A tacholess ridge extraction method enables robust rotational-frequency estimation under noise and multi-component interference.
- UDR-CWAE improves latent representation through MMD-based distribution regularisation.

**What are the implications of the main findings?**

- Single-rotation-cycle samples reduce speed-induced structural inconsistency in vibration signals.
- Uncertainty-weighting strategy enhances training stability and cross-condition generalisation.

**Abstract:**

Cross-condition fault diagnosis of rotating machinery remains challenging under variable speed and load because vibration signals have strong non-stationarity and exhibit distribution shifts across operating conditions. To address this problem, a tacholess diagnosis framework combining time–frequency ridge extraction with an uncertainty-guided distribution-regularised convolutional Wasserstein autoencoder (UDR-CWAE) is proposed. Firstly, instantaneous rotational frequency is estimated directly from vibration signals using harmonic-amplitude-based ridge initialisation, edge-constrained search, and cost-function-based tracking. Then, the estimated rotational frequency is integrated to construct single-rotation-cycle vibration samples, which are normalised to reduce the discrepancy of amplitude scale among samples. Finally, UDR-CWAE regularises the aggregated latent distribution using maximum mean discrepancy, while uncertainty weighting adaptively balances reconstruction, distribution-regularisation, and classification losses. Cross-condition experiments on the Ottawa bearing dataset and the SQI test-rig dataset achieved classification accuracies of 99.98% and 99.23%, respectively. These results demonstrate that the proposed framework provides accurate tacholess speed estimation and robust fault recognition under unseen rotational-frequency conditions.

## 1. Introduction

With the widespread application of equipment such as centrifugal compressors, machine tools, and wind turbines, the stability and reliability of rotating machinery have become directly associated with production efficiency and operational safety [1,2,3]. As an important carrier of the dynamic state of rotating machinery, vibration signals have been widely used for fault monitoring and diagnosis [4]. However, in practical industrial environments, machinery operation is typically affected by multiple factors, including rotational speed, load, medium disturbances, and control strategies [5]. As a result, vibration responses often exhibit pronounced non-stationarity and operating-condition dependence. Therefore, achieving the stable and reliable fault identification of rolling bearings under complex variable operating conditions remains an urgent challenge in the fields of intelligent monitoring and predictive maintenance [6,7].

Variable rotational speed is one of the primary factors affecting the feature representation of vibration signals in rotating machinery. When the rotational speed varies with time, fault characteristic frequencies associated with the rotational-frequency drift accordingly, leading to the broadening, overlap, or even noise-induced masking of fault-related components in conventional frequency spectra [8,9,10]. Order tracking can effectively mitigate the influence of speed fluctuations through angular-domain resampling [11,12]. However, such methods generally require tachometers, encoders, or key-phase signals to provide angular synchronisation information. In many industrial scenarios, additional speed-measurement devices are not always available owing to constraints related to equipment structure, installation space, safety requirements, and maintenance cost. Therefore, estimating the rotational frequency under tacholess conditions using vibration sensor signals alone constitutes a key issue in improving the engineering applicability of intelligent diagnostic methods for rotating machinery.

Time–frequency analysis provides an effective means for tacholess fault diagnosis [13,14]. By mapping one-dimensional vibration signals onto a two-dimensional time–frequency plane, it enables the temporal evolution of non-stationary frequency components to be characterised, and further allows the extraction of time–frequency ridges associated with the rotational frequency and its harmonics. However, practical vibration signals often contain multiple order components, fault-induced impulsive components, and background noise. Consequently, the rotational-frequency component is not always the most energetically dominant component in the time–frequency representation [15]. Recent studies have investigated noise-robust fault diagnosis through adaptive signal decomposition and advanced feature-learning architectures. For example, Zhu et al. [16] integrated adaptive tuning feature mode decomposition with a synergy graph-enhanced Transformer to suppress noise-induced redundant information and improve fault-feature representation under noisy conditions. Nevertheless, robust estimation of instantaneous rotational frequency from vibration signals alone remains challenging when multiple spectral components and spurious ridges coexist. Under fault conditions such as misalignment, looseness, or impact-related defects, the second harmonic or higher-order harmonics may be more prominent than the fundamental frequency. Therefore, ridge extraction based solely on local peak searching [17,18,19] is prone to ridge mis-selection. Although conventional cost-function-based ridge estimation methods [20,21] incorporate ridge continuity, their results remain susceptible to the predefined search range and the presence of spurious ridges.

In addition to the variable rotational speed, load variation can also alter the amplitude scale, impact response, and frequency-domain energy distribution of vibration signals. When a diagnostic model is trained under one operating condition and tested under another unseen condition, samples belonging to the same fault category may exhibit pronounced shifts in the feature space. Moreover, practical diagnostic datasets may also suffer from class imbalance and heterogeneous fault information across different representation domains. Zhu et al. [22] addressed imbalanced bearing fault diagnosis by combining sample augmentation with multidomain feature fusion, exploiting complementary information from the time, frequency, and sparse domains to improve minority-class discrimination and diagnostic robustness. These advances highlight the benefits of robust feature enhancement, sample balancing, and multidomain representation learning. However, when diagnostic models are trained under known operating conditions and subsequently applied to unseen speed or load conditions, the distribution discrepancy between source- and target-condition samples remains a major obstacle to reliable fault recognition. Conventional discriminative models, such as Convolutional Neural Networks (CNNs) [23,24] and Support Vector Machines (SVMs) [25,26], usually rely on operating-condition-related features learned from the training data, and therefore struggle to ensure robust fault identification across varying operating conditions. By contrast, generative models, such as Variational Autoencoders (VAEs) [27,28], Generative Adversarial Networks (GANs) [29,30] and diffusion models [31,32], perform signal reconstruction and latent-space modelling simultaneously, which facilitates the learning of deep features with improved generalisation capability. Nevertheless, classical VAEs may suffer from a trade-off between reconstruction and classification objectives in multi-task learning, while the KL-divergence constraint may limit the representational capacity of the latent space. Hence, there is an urgent need to introduce more flexible generative structures and adaptive task weighting mechanisms to enhance the generalisation performance of diagnostic models under unknown operating conditions.

To address the aforementioned issues, this study proposes an intelligent fault-diagnosis method for rolling bearings that combines time–frequency ridge extraction with an uncertainty-guided distribution-regularised convolutional Wasserstein autoencoder. This method first utilises time–frequency spectral structure information as a guiding framework and locates an initial point of the ridge by searching for the peak of the sum of harmonic amplitudes. It then combines Canny edge detection optimised via bilateral filtering to adaptively delimit the search domain and relies on ridge detection with an improved cost function and a cubic spline interpolation strategy to achieve automatic rotational-frequency extraction without external speed sensors. Based on the estimated rotational frequency, single-rotation-cycle samples are then constructed, and normalisation is applied to mitigate amplitude shifts induced by load variation. Finally, an uncertainty-guided distribution-regularised convolutional Wasserstein autoencoder model (UDR-CWAE) is developed, in which maximum mean discrepancy (MMD) [33] regularisation is employed to impose distributional constraints on the latent space. An uncertainty-weighting strategy is further introduced to adaptively balance the reconstruction, distribution regularisation, and classification tasks, thereby enhancing the generalisation capability of the model under unseen operating conditions.

The main contributions of this study are as follows:

(1) A tacholess ridge extraction method is proposed by integrating harmonic amplitude summation search, bilateral-filter-based Canny edge detection, and cost-function-based ridge estimation. The proposed method is capable of extracting continuous and smooth rotational-frequency ridges under multi-component interference and background noise.
(2) A single-rotation-cycle sample representation is constructed based on the estimated rotational frequency, such that each sample corresponds to one complete rotation of the machinery. This representation helps reduce the structural inconsistency of samples caused by variable rotational speed.
(3) A UDR-CWAE model is designed for the cross-condition fault diagnosis of rolling bearings, in which MMD is employed to constrain the latent-space distribution. This design alleviates the insufficient latent representation capacity caused by the KL-divergence constraint in conventional VAEs.
(4) An uncertainty-weighting strategy is introduced to adaptively coordinate the reconstruction loss, MMD regularisation term, and classification loss. This strategy avoids the manual specification of loss weights, thereby improving training stability and enhancing the cross-condition generalisation performance of the model.

The remainder of this paper is organised as follows. Section 2 presents the rotational-frequency estimation method under tacholess conditions and the construction of single-rotation-cycle samples. Section 3 introduces the uncertainty-guided distribution-regularised convolutional Wasserstein autoencoder for cross-condition fault diagnosis. Section 4 reports the experimental validation results based on two fault datasets. Finally, Section 5 summarises the main conclusions of this study.

## 2. Tacholess Rotational-Frequency Estimation and Cycle-Synchronous Sample Construction

**Figure 1:**
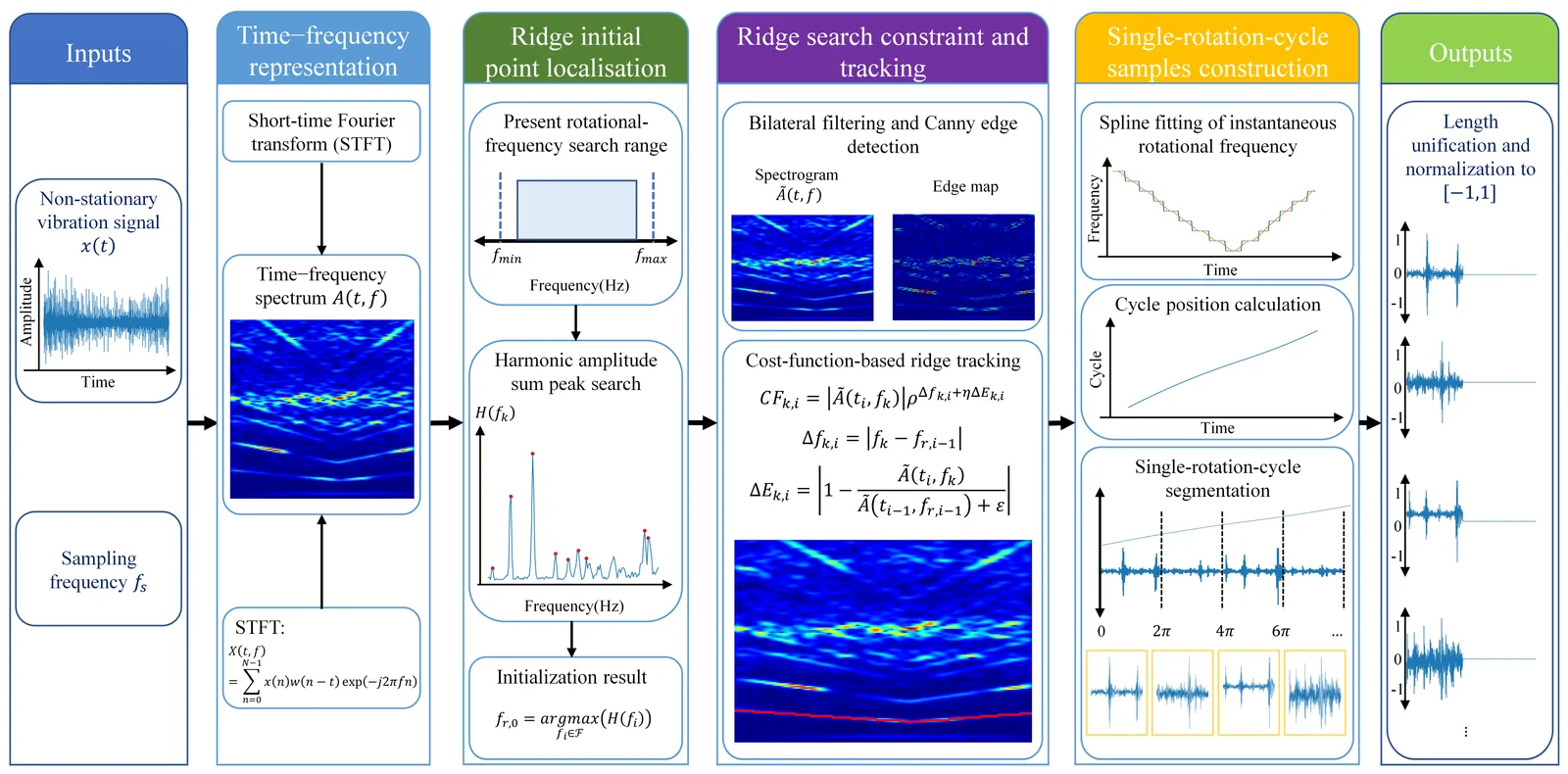
Framework of tacholess rotational-frequency extraction and cycle-synchronised sample construction.

Within the core of time–frequency ridge extraction lies the precise localization of the peak sequences corresponding to the rotational-frequency components. When a signal comprises solely a signal-order component, only one ridge manifests in the time–frequency spectrum, rendering the corresponding ridge extraction procedure comparatively straightforward. However, vibration signals from variable-speed rotating machinery under practical operating conditions typically comprise multi-order components and are generally corrupted by background noise. To address the above challenges, this study proposes a tacholess rotational-frequency extraction method that integrates harmonic amplitude summation and peak searching, bilateral-filter-based Canny edge detection, and cost-function-based ridge estimation. The overall framework of the proposed method is illustrated in Figure 1. Specifically, the method first determines the initial ridge position by exploiting the harmonic relationship between the rotational frequency and its harmonics. The search region is subsequently constrained using edge detection, and a rotational-frequency ridge is obtained through cost-function-based ridge estimation. Finally, cubic spline interpolation is applied to fit a continuous and smooth instantaneous rotational-frequency curve, based on which single-rotation-cycle samples are constructed.

### 2.1. Ridge Initial Point Localisation via Harmonic Amplitude Summation

To characterise the temporal evolution of frequency components in vibration signals, the short-time Fourier transform (STFT) is first applied to the original signal. For a discrete vibration signal $x\left(n\right)$, the STFT can be expressed as

(1) $$X\left(t,f\right)=\sum_{n=0}^{N-1}x\left(n\right)w\left(n-t\right)\exp\left(-j2\pi fn\right),$$

where $w\left(n-t\right)$ is the Hann window function with a length equal to the sampling frequency $f_{s}$. The overlap of $0.95f_{s}$ for STFT is adopted in this study. The corresponding time–frequency amplitude spectrum is given by

(2) $$A\left(t,f\right)=\left|X\left(t,f\right)\right|,$$

In the time–frequency plane, the rotational frequency and its harmonics appear as energy ridges with continuously varying trajectories. Ideally, the rotational-frequency ridge can be expressed as:

(3) $$r=\left\{f_{r}\left(t_{1}\right),f_{r}\left(t_{2}\right),\ldots ,f_{r}\left(t_{T}\right)\right\},$$

where T represents the total number of time slices in the time–frequency spectrum.

For practical vibration signals, the rotational-frequency component may not correspond to the dominant amplitude peak in the low-frequency region. Accordingly, the proposed method does not identify the rotational frequency by directly selecting an isolated maximum peak. Instead, initial localisation is conducted by exploiting the overall energy distribution associated with the rotational frequency and its harmonic components.

Let the candidate rotational-frequency set be defined as

(4) $$F=\left\{f_{min}\leq f_{i}\leq f_{max}\right\},$$

where $f_{min}$ and $f_{max}$ are the lower and upper limits of the search range of candidate frequency conversion, respectively. In practical implementation, $f_{max}$ is selected with a sufficiently large margin relative to the expected maximum rotational frequency. A search upper bound approximately three times or more than the expected rotational-frequency range was considered adequate for avoiding premature restriction of candidate frequencies. Accordingly, $f_{min}$ and $f_{max}$ are set to 0 and 100 Hz, respectively, in the present experiments. For any candidate frequency $f_{i}$, the summation of its first K harmonics is defined as follows:

(5) $$H\left(f_{i}\right)=\sum_{k=1}^{K}A\left(f_{k}\right)\;f_{k}=kf_{i}$$

where K determines the number of low-order harmonics involved in ridge initialisation. A small K may provide insufficient harmonic information when the fundamental component is weak, whereas an excessively large K may introduce unrelated high-frequency components and noise. The value of K is set to three in this study.

Considering that the time–frequency spectrum has finite frequency resolution, the actual harmonic frequency points may not strictly fall at $f_{k}$. Therefore, the frequency tolerance Δf is introduced in this paper, and the corresponding harmonic amplitude is searched in the neighbourhood:

(6) $$A\left(f_{k}\right)=\underset{f\in \left[f_{k}-\Delta f,f_{k}+\Delta f\right]}{\max}A\left(f\right)\;\Delta f=c_{f}\frac{f_{s}}{N}$$

where $f_{s}$ denotes the sample frequency, $c_{f}$ is generally selected within the range of 1–3 according to the STFT frequency resolution, and $c_{f}=2$ was used in all experiments in this study. Finally, the initial rotational-frequency estimate can be expressed as

(7) $$f_{r,0}=\underset{f_{i}\in F}{\mathrm{argmax}}\left(H\left(f_{i}\right)\right)$$

This strategy essentially exploits the harmonic multiple-frequency relationship between the rotational frequency and its harmonics, selecting the candidate frequency with the most prominent overall harmonic energy as the initial rotational-frequency point. Compared with single-peak searching, the proposed strategy reduces the influence of locally dominant interference components on initial localisation and is particularly suitable for fault conditions characterised by a prominent second harmonic, enhanced harmonic components, or low-frequency interference.

### 2.2. Ridge Search-Range Constraint and Tracking

After the initial rotational-frequency position is determined, the complete ridge needs to be tracked along the time direction. However, in a multi-component time–frequency representation, different order components may approach or even intersect with each other, while noise and locally dominant energy components may also cause abrupt ridge jumps. To avoid erroneous connection to irrelevant components during ridge tracking, this study treats the time–frequency amplitude spectrum as a two-dimensional image and introduces bilateral-filter-based Canny edge detection to constrain the ridge search range.

Firstly, the time–frequency amplitude spectrum $A\left(t,f\right)$ is processed by bilateral filtering:

(8) $$\tilde{A}\left(t,f\right)=\frac{1}{W_{p}}\sum_{\left(i,j\right)\in \Omega }A\left(i,j\right)\exp\left(-\frac{{\left\|\left(t,f\right)-\left(i,j\right)\right\|}^{2}}{2\sigma_{s}^{2}}-\frac{{\left\|A\left(t,f\right)-A\left(i,j\right)\right\|}^{2}}{2\sigma_{r}^{2}}\right),$$

where Ω is the local neighbourhood window, $\sigma_{s}$ is the standard deviation of spatial domain, $\sigma_{r}$ is the standard deviation of amplitude domain, and $W_{p}$ represents the normalised coefficient. Bilateral filtering can suppress the background noise of the time–frequency map while preserving the ridge edge structure.

Subsequently, the gradient of the filtered time–frequency map $\tilde{A}\left(t,f\right)$ is calculated by the Sobel operator. The gradients in the time and frequency directions are as follows:

(9) $$G_{t}=\tilde{A}*S_{t}\;G_{f}=\tilde{A}*S_{f},$$

where $S_{t}$ and $S_{f}$ are the Sobel difference operators in the time and frequency directions, respectively. The gradient magnitude and gradient direction are as follows:

(10) $$G\left(t,f\right)=\sqrt{G_{t}^{2}+G_{f}^{2}}$$

(11) $$\Theta \left(t,f\right)=\arctan\left(\frac{G_{f}}{G_{t}}\right)$$

Based on the above procedure, the thinned edge map $E\left(t,f\right)$ is obtained by applying non-maximum suppression. Specifically, after the ridge point $f_{r}\left(t_{i-1}\right)$ at the $t_{i-1}$-th time slice has been determined, the edge responses in the subsequent time slice are examined. The nearest valid edge positions below and above the continuation region of the current ridge are denoted by $f_{t_{i}}^{L}$ and $f_{t_{i}}^{U}$, respectively. The candidate search region $R_{t_{i}}$ for the next time slice is then defined as

(12) $$f_{t_{i}}^{L}=\max\left\{f<f_{r}\left(t_{i-1}\right)|E\left(t_{i},f\right)>0\right\},\;f_{t_{i}}^{U}=\min\left\{f>f_{r}\left(t_{i-1}\right)|E\left(t_{i},f\right)>0\right\},\;R_{t_{i}}=\left\{f|f_{t_{i}}^{L}\leq f\leq f_{t_{i}}^{U}\right\}$$

With this constraint, subsequent ridge tracking is performed only within local regions characterised by distinct edge structures and frequency continuity, thereby reducing interference from spurious ridges and irrelevant components.

After the candidate search region is determined, an improved cost-function-based ridge estimation algorithm is employed in this study to extract the complete rotational-frequency ridge. An ideal rotational-frequency ridge should satisfy three conditions simultaneously. First, the candidate point should have a relatively high amplitude in the time–frequency representation, ensuring that the ridge path passes through the main energy-concentration region. Second, the frequency difference between the candidate points and the ridge point in the previous time slice should be as small as possible, so as to maintain temporal continuity of the ridge trajectory. Third, the relative amplitude variation between the candidate points and the ridge point in the previous time slice should also be minimised, thereby preventing abrupt ridge jumps caused by locally strong noise, transient impulses, or spurious peaks.

For a candidate frequency point $f_{k}$ in the current time slice $t_{i}$, the frequency variation is defined as

(13) $$\Delta f_{k,i}=\left|f_{k}-f_{r,i-1}\right|$$

The relative amplitude variation between adjacent time slices is defined as

(14) $$\Delta E_{k,i}=\left|1-\frac{\tilde{A}\left(t_{i},f_{k}\right)}{\tilde{A}\left(t_{i-1},f_{r,i-1}\right)+\varepsilon }\right|,$$

where ε is a small positive constant introduced to avoid division by zero. The cost function $CF_{k}$ for the candidate frequency point $f_{k}$ is defined as

(15) $$CF_{k,i}=\left|\tilde{A}\left(t_{i},f_{k}\right)\right|\rho^{\Delta f_{k,i}+\eta \Delta E_{k,i}},$$

where $\rho \in \left(0,1\right)$ is the attenuation coefficient, and η is the amplitude-variation penalty factor. The proposed cost function consists of an amplitude-enhancement term and a continuity-penalty term. A larger value of $\left|\tilde{A}\left(t_{i},f_{k}\right)\right|$ indicates that the candidate point is more likely to lie on the true ridge. A smaller $\Delta f_{k,i}$ suggests smoother frequency variation between the current candidate point and the preceding ridge point, whereas a smaller $\Delta E_{k,i}$ indicates better amplitude continuity between adjacent time slices. Since $\rho \in \left(0,1\right)$, an increase in either frequency jumping or abrupt amplitude variation reduces the exponential decay term, thereby decreasing the probability that the corresponding candidate point will be selected as a ridge point. In this study, the attenuation coefficient ρ and the amplitude-variation penalty factor η were set to 0.95 and 5, respectively. The attenuation coefficient ρ controls the overall strength of the continuity constraint in ridge tracking. A relatively large value of ρ avoids excessive suppression of legitimate frequency variations under variable-speed conditions, while still penalising abrupt frequency jumps and amplitude fluctuations caused by noise or spurious spectral components. The parameter *η* determines the relative contribution of amplitude continuity to the cost function. Setting *η* = 5 increases the sensitivity of the cost function to abrupt amplitude variations, thereby reducing the likelihood of the ridge being incorrectly attracted to transient high-energy components.

Therefore, the optimal ridge frequency point at the i-th time slice can be expressed as

(16) $$f_{r,i}=\underset{f_{k}\in R_{t_{i}}}{\mathrm{argmax}}\left(CF_{k,i}\right)$$

In the implementation, the search starts from the identified initial rotational-frequency point and proceeds iteratively in both the forward and backward temporal directions. For each time slice, the candidate search region $R_{i}$ is first determined according to the edge-detection result and the previously extracted ridge point. The cost function values of all candidate frequency points within this region are then calculated, and the point with the maximum cost value is selected as the current ridge point. By repeating this procedure, the complete rotational-frequency ridge can be obtained:

(17) $$\hat{f_{r}}\left(t_{i}\right)=f_{r,i},\;i=1,2,\ldots ,T$$

### 2.3. Single-Rotation-Cycle Samples Construction

**Figure 2:**
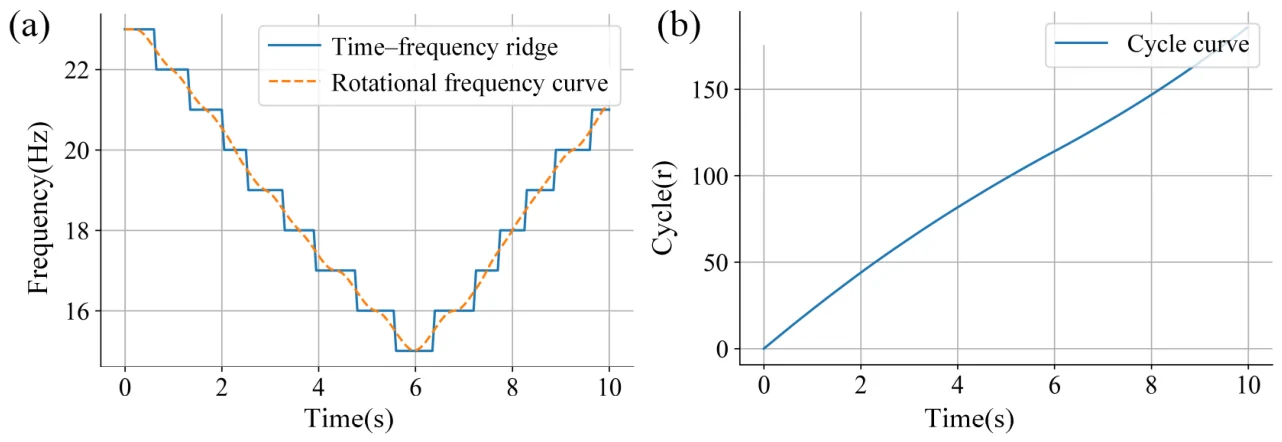
Extracted rotational-frequency curve and period curve of rotary machinery: (**a**) instantaneous rotational frequency; (**b**) cycle curve.

The instantaneous rotational-frequency curve $\hat{f_{r}}\left(t\right)$ obtained from time–frequency ridge extraction still contains local fluctuations and estimation errors. To obtain a smoother rotational-frequency curve ${\bar{f}}_{r}\left(t\right)$ with a temporal resolution consistent with that of the vibration signal $x\left(n\right)$, a cubic spline function is employed in this study. The fitting result is shown in Figure 2a. According to the relationship between instantaneous rotational frequency and rotational cycle, the cumulative number of rotation cycles $C\left(t\right)$ can be obtained by integrating ${\bar{f}}_{r}\left(t\right)$:

(18) $$C\left(t\right)=\int_{0}^{t}{\bar{f}}_{r}\left(\tau \right)d\tau ,$$

since ${\bar{f}}_{r}\left(t\right)$ is expressed in Hz, an increment of one in $C\left(t\right)$ corresponds to one complete revolution of the rotating machinery. In the discrete implementation, the cumulative cycle number is calculated numerically from the sampled rotational-frequency sequence. The curve of the rotation cycles $C\left(t\right)$ is shown in Figure 2b.

where $f_{s}$ is the sampling frequency of vibration signal $x\left(n\right)$. When $C\left(t\right)$ increases by one, the machinery completes one full rotation cycle. Therefore, the m-th single-rotation-cycle sample can be represented as:

(19) $$x_{m}=\left\{x\left(t\right)|m-1\leq C\left(t\right)<m\right\},$$

**Figure 3:**
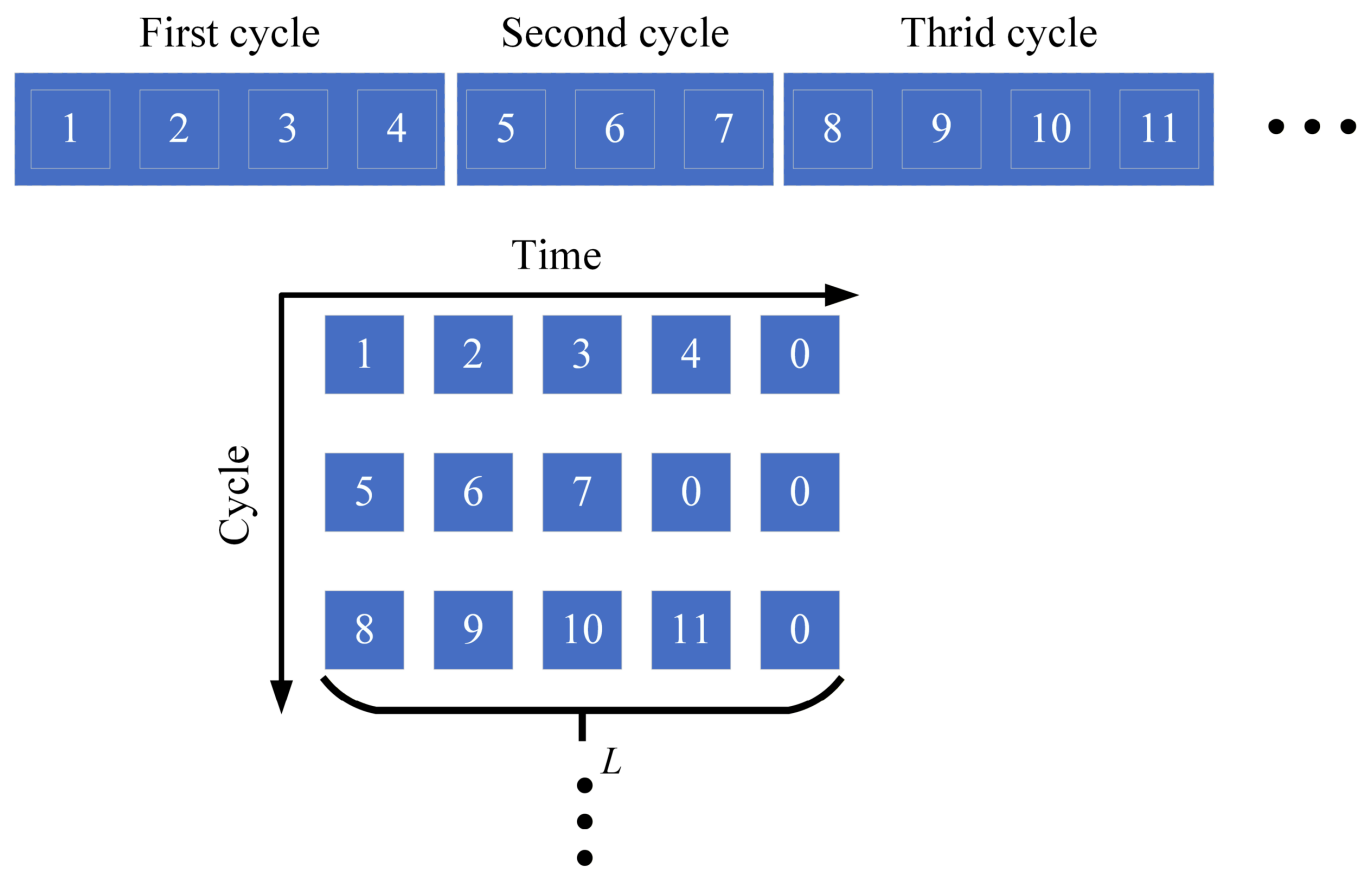
Schematic diagram of cycle-synchronous sliding-window sample extraction.

Since the machinery operates under variable-speed conditions, the number of sampling points contained in a single rotation cycle varies with the instantaneous rotational frequency. For the *m*-th cycle, its approximate length satisfies $N_{m}\approx f_{s}/f_{r,m}$. Therefore, a fixed input length L is introduced to enable batch processing by the neural network. For a cycle sample with $N_{m}<L$, the original vibration samples are retained and zeros are appended to obtain a length of L. Zero-padding does not alter the measured portion of the vibration waveform or introduce additional physical fault components. The padded region only provides dimensional consistency for the network input. For samples with $N_{m}>L$, the complete cycle is resampled to L points by linear interpolation. The cycle-synchronous sliding-window sample extraction procedure is illustrated in Figure 3.

Subsequently, to mitigate amplitude-scale discrepancies caused by different loads and measurement locations, each cycle sample is normalised as

(20) $$x_{m}^{norm}=2\frac{{\tilde{x}}_{m}-\min\left({\tilde{x}}_{m}\right)}{\max\left({\tilde{x}}_{m}\right)-\min\left({\tilde{x}}_{m}\right)}-1,$$

Through the above procedure, the original non-stationary vibration signals are transformed into single-rotation-cycle samples with rotational-frequency-synchronous characteristics. However, under different loads or operating conditions, samples belonging to the same fault category may still exhibit discrepancies in amplitude scale and deep feature distribution, even after cycle-synchronous processing. Therefore, it remains necessary to further construct a diagnostic model with distribution-constraining capability and cross-condition generalisation ability.

## 3. Cross-Condition Fault Diagnosis Model Based on UDR-CWAE

Load fluctuation alters the amplitude scale and frequency-domain distribution characteristics of vibration signals, leading to differences in the data distribution between the training and test sets, namely the phenomenon of distribution drift. This constitutes one of the core causes underlying the difficulty of cross-operating-condition fault identification in rotating machinery. To improve fault-identification accuracy and generalisation capability under distribution discrepancies between training and testing operating conditions, this study develops an uncertainty-guided distribution-regularised convolutional Wasserstein autoencoder, termed UDR-CWAE. The proposed model is built upon a convolutional autoencoder framework. The reconstruction task is employed to preserve the structural information of the input signals, while latent-space distribution regularisation is introduced to constrain the deep feature distributions of samples from different operating conditions. In addition, the classification task is incorporated to enhance the fault-discriminative capability of the latent variables. Meanwhile, a homoscedastic uncertainty-based weighting strategy is adopted to adaptively coordinate the contributions of the reconstruction loss, distribution regularisation term, and classification loss.

### 3.1. Architecture of the UDR-CWAE Model

**Figure 4:**
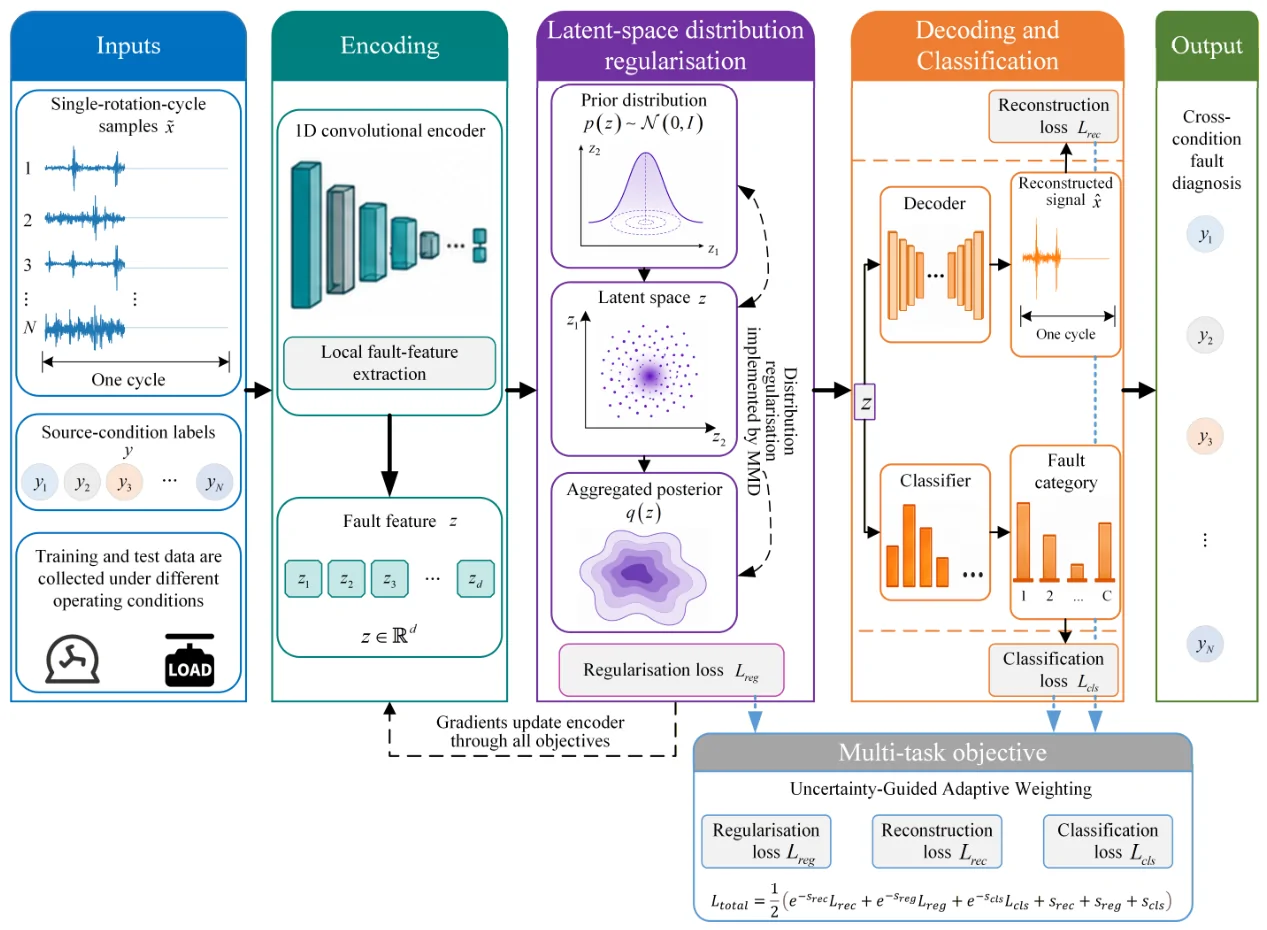
Framework of the proposed UDR-CWAE for cross-condition fault diagnosis.

The framework of the proposed UDR-CWAE model is shown in Figure 4. UDR-CWAE mainly consists of three components: an encoder, a decoder, and a classifier. The encoder is composed of multiple one-dimensional convolutional layers, normalisation layers, and nonlinear activation layers, and is used to extract low-dimensional latent features from cycle-synchronous vibration samples. The decoder adopts a transposed convolutional structure to map the latent variables back to the input space and generate reconstructed samples. The classifier predicts the fault category based on the latent variables.

The basic idea of UDR-CWAE is to obtain latent-space representations through the encoder, ensure the input-signal reconstruction capability of the latent variables through the decoder, enhance their fault-discriminative capability through the classifier, and constrain their overall distribution through distribution regularisation. In this way, the stability of the model under cross-condition scenarios can be improved.

### 3.2. Latent-Space Distribution Regularisation

Conventional VAE generally employ the Kullback–Leibler (KL) divergence to constrain the discrepancy between the posterior distribution and the prior distribution. However, when modelling complex vibration signals, an excessively strong KL constraint may reduce the expressive capability of the latent variables and may even lead to posterior collapse. Wasserstein autoencoders (WAEs) instead focus on the global matching between the encoded aggregated latent distribution and the prior distribution. Compared with sample-wise posterior constraints, this formulation provides a more flexible latent-space representation capability.

Let the real data distribution be denoted as $p_{d}\left(x\right)$, The aggregated posterior distribution $q_{z}$ induced by the encoder can then be expressed as

(21) $$q_{z}\left(z\right)=\int q\left(z|x\right)p_{d}\left(x\right)dx,$$

The Wasserstein distance between the data distribution and the generated distribution can be formulated as a reconstruction problem subject to the constraint that the aggregated posterior distribution of the encoded latent variables matches a predefined latent prior. In practice, this constrained problem is relaxed as

(22) $$L_{WAE}=E_{p_{d}\left(x\right)}E_{q\left(z|x\right)}\left({\left\|x-\hat{x}\right\|}_{2}^{2}\right)+\lambda D_{z}\left(q_{z},p_{z}\right),$$

where $D_{z}$ denotes a distribution discrepancy measure and λ controls the strength of the distribution regularisation of latent features. In the implementation, $D_{z}$ is instantiated using maximum mean discrepancy (MMD).

Let the prior distribution in the latent space be defined as a standard normal distribution, namely $p_{z}\left(z\right)=N\left(0,1\right)$. To regularise the latent distribution, a distribution regularisation term is introduced to minimise the discrepancy between $q_{z}\left(z\right)$ and $p_{z}\left(z\right)$. The MMD is defined as follows:

(23) $$MMD^{2}\left(q_{z},p_{z}\right)={\left\|E_{z\sim q_{z}}\left(\varphi \left(z\right)\right)-E_{z^{\prime }\sim p_{z}}\left(\varphi \left(z^{\prime }\right)\right)\right\|}_{H}^{2},$$

where $\varphi \left(\cdot \right)$ denotes the mapping function into the reproducing kernel Hilbert space (RKHS), and H denotes the corresponding function space. For a mini-batch of samples, let ${\left\{z_{i}\right\}}_{i=1}^{n}$ denote the set of latent variables output by the encoder, and let ${\left\{z_{i}^{\prime }\right\}}_{i=1}^{n}$ denote the set of latent variables sampled from the prior distribution. The sample-based estimation of MMD can then be formulated as follows:

(24) $$L_{reg}=\frac{1}{n^{2}}\sum_{i\neq j}k\left(z_{i},z_{j}\right)+\frac{1}{n^{2}}\sum_{i\neq j}k\left(z_{i}^{\prime },z_{j}^{\prime }\right)-\frac{2}{n^{2}}\sum_{i=1}^{n}\sum_{j=1}^{n}k\left(z_{i},z_{j}^{\prime }\right),$$

where $k\left(\cdot \right)$ denotes the kernel function. In this study, the Gaussian kernel function is employed:

(25) $$k\left(z_{i},z_{j}\right)=\sum_{m=1}^{M}\exp\left(-\frac{{\left\|z_{i}-z_{j}\right\|}^{2}}{2\sigma_{m}^{2}}\right),$$

A multi-scale Gaussian radial basis function kernel is adopted, with the bandwidth set

(26) $${\left\{\sigma_{m}\right\}}_{m=1}^{5}=\left\{1,\;2,\;4,\;8,\;16\right\},$$

The multi-scale kernel is used to account for latent-distribution discrepancies at different characteristic scales. The same bandwidth set is used throughout the experiments. By introducing $L_{reg}$, the aggregated latent distribution induced by the encoder is regularised towards the predefined prior distribution, thereby promoting a more regularised latent representation. $L_{reg}$ does not directly align the source and target domains, since target-condition samples are not used during model training.

### 3.3. Uncertainty-Guided Adaptive Weighting for Multi-Task Losses

UDR-CWAE involves both reconstruction and classification tasks. The reconstruction task requires the model to recover the original input signal from the latent variables, thereby encouraging the latent variables to retain the main structural information of the input samples. The reconstruction loss is defined as follows:

(27) $$L_{rec}=\frac{1}{N}\sum_{n=1}^{N}{\left\|x_{n}^{norm}-{\hat{x}}_{n}\right\|}_{2}^{2},$$

where N is the number of samples, $x_{n}^{norm}$ denotes the normalised input sample, and ${\hat{x}}_{n}$ denotes the sample reconstructed by the decoder.

The classification task requires the latent variables to possess discriminative capability for fault categories. The cross-entropy loss is adopted as the classification loss

(28) $$L_{cls}=-\frac{1}{N}\sum_{n=1}^{N}\sum_{c=1}^{C}y_{n,c}\log\left({\hat{y}}_{n,c}\right),$$

where C denotes the number of fault categories, $y_{n,c}$ denotes the true label, and ${\hat{y}}_{n,c}$ denotes the predicted probability of the model.

If the three types of losses are directly combined using fixed weights, the total loss can be written as follows:

(29) $$L_{total}=\alpha L_{rec}+\beta L_{reg}+\gamma L_{cls},$$

where α, β, and γ are manually specified weighting coefficients. However, these weights have a considerable influence on the training performance of the model and often need to be readjusted for different datasets and operating conditions.

Therefore, an uncertainty-weighting strategy is further introduced in this study to achieve adaptive coordination of the multi-task losses. The core idea of uncertainty weighting is to regard the loss of each task as an observation model with noise uncertainty and to automatically adjust the contribution of each loss term to the overall objective by learning the task uncertainty. For the reconstruction and classification objectives, the learnable parameters retain their conventional uncertainty-inspired interpretation. For the MMD regularisation term, it acts as a learnable adaptive loss-scaling parameter that adaptively controls the contribution of latent-distribution regularisation relative to the other objectives. This formulation prevents any single loss component from dominating the joint optimisation while avoiding manually selected fixed weights.

Let the uncertainties corresponding to the reconstruction task, distribution regularisation task, and classification task be denoted as $\sigma_{rec}^{2}$, $\sigma_{reg}^{2}$, and $\sigma_{cls}^{2}$, respectively. The total loss can then be expressed as follows:

(30) $$L_{total}=\frac{1}{2\sigma_{rec}^{2}}L_{rec}+\frac{1}{2\sigma_{reg}^{2}}L_{reg}+\frac{1}{2\sigma_{cls}^{2}}L_{cls}+\log\sigma_{rec}+\log\sigma_{reg}+\log\sigma_{cls},$$

To improve numerical stability, let $s_{i}=\log\sigma^{2}$. Equation (27) can then be rewritten as follows:

(31) $$L_{total}=\frac{1}{2}\left(e^{-s_{rec}}L_{rec}+e^{-s_{reg}}L_{reg}+e^{-s_{cls}}L_{cls}\right)+\frac{1}{2}\left(s_{rec}+s_{reg}+s_{cls}\right),$$

where $s_{rec}$, $s_{reg}$, and $s_{cls}$ are all learnable parameters. When the loss of a task is large or its training process is unstable, the corresponding uncertainty parameter increases, and the model automatically reduces the weight assigned to that task loss. Conversely, when a task becomes more stable, its relative weight increases. The learnable parameters are updated together with the network parameters through back-propagation, allowing the relative weights of the reconstruction, MMD regularisation, and classification losses to be determined adaptively during training rather than manually specified in advance.

Through the uncertainty-weighting strategy, UDR-CWAE not only learns the reconstructed structure of the input samples but also maintains the regularity of the latent-space distribution and enhances the discriminative capability for fault categories. This multi-task collaborative mechanism contributes to improving the stability and generalisation performance of the model under unknown operating conditions.

### 3.4. Model Training and Diagnosis Procedure

In the model training stage, the cycle-synchronised sample $x_{m}^{norm}$ constructed in Section 2 is first fed into the encoder to obtain the latent variable $z_{m}$. Subsequently, the decoder reconstructs the input signal from $z_{m}$, while the classifier outputs the fault-category probability according to $z_{m}$. Meanwhile, $z^{\prime }\sim N\left(0,I\right)$ is sampled from the standard normal distribution and used to calculate the latent-space distribution regularisation loss.

The training objective of the model is formulated as follows:

(32) $$\theta^{*},\phi^{*},\varphi^{*}=\underset{\theta ,\phi ,\varphi }{\mathrm{argmin}}\;L_{total},$$

where θ, ϕ, and φ denote the parameters of the encoder, decoder, and classifier, respectively.

In the testing stage, only the encoder and classifier are required to perform fault identification. For a test sample $x_{t}^{norm}$ under an unknown operating condition, the diagnostic result is given by

(33) $${\hat{y}}_{t}=\underset{c}{\mathrm{argmax}}\;C_{\varphi }{\left(E_{\theta }\left(x_{t}^{norm}\right)\right)}_{c},$$

where ${\hat{y}}_{t}$ is the fault category predicted by the model. Since the model simultaneously performs cycle-synchronised representation learning, latent-space distribution constraint, and uncertainty-weighted multi-task optimisation during training, it can maintain favourable recognition performance when distribution discrepancies exist between the training and testing operating conditions.

The detailed architecture of the proposed UDR-CWAE is summarised in Table 1. the encoder consists of three one-dimensional convolutional blocks with channel numbers of 32, 64, and 128, respectively, followed by full connected layers that map the extracted features into a 32-dimensional latent space. The decoder employs a symmetric transposed convolutional structure to reconstruct the input, while the classifier maps the latent representation regularised by MMD to the corresponding fault category. The model was trained using the AdamW optimiser for 30 epochs, with a mini-batch size of 64 and an initial learning rate of $10^{-3}$. The weight-decay coefficient was set to $10^{-4}$, and the latent-space dimension was fixed at 32. The dropout probability was set to 0.2 for the corresponding encoder and classifier layers. All experiments were conducted using the same training configuration unless otherwise specified.

## 4. Experimental Results

To verify the reliability and generalisation capability of the proposed method, two independent bearing fault datasets are selected for experimental validation. The first dataset is the publicly available variable-speed bearing dataset from the University of Ottawa [34], while the second dataset is the bearing fault dataset collected under time-varying operating conditions using the SQI bearing fault simulation test bench.

### 4.1. Case Analysis on the Ottawa Bearing Fault Data

#### 4.1.1. Introduction to Ottawa Bearing Fault Data

**Figure 5:**
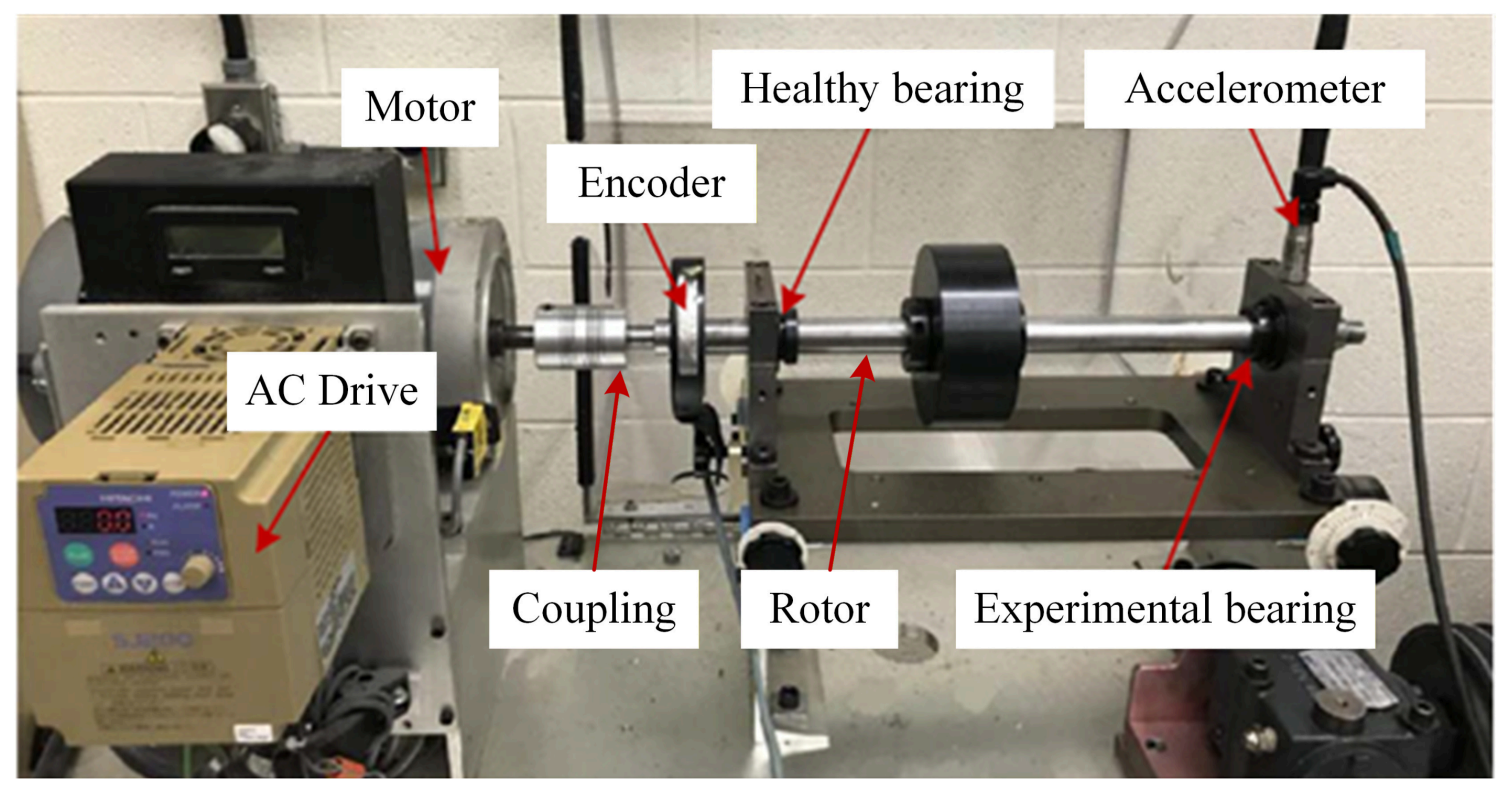
Experimental platform diagram of the SpectraQuest machinery fault simulator.

The Ottawa bearing fault dataset was collected using a SpectraQuest machinery fault simulator (SpectraQuest, Inc., Richmond, VA, USA). The experimental platform, shown in Figure 5, mainly consists of a motor, an AC drive, an encoder, a coupling, a rotor, and bearings. The rig is driven by a 1 HP AC induction motor, and the rotational speed is regulated using a programmable AC drive. Two ER16K ball bearings are installed on the experimental platform. The left bearing is maintained in a healthy condition, whereas the right bearing serves as the test bearing and is replaced by bearings with different fault conditions in different experiments. A 623C01 accelerometer is mounted on the test bearing housing to acquire vibration acceleration signals, and an EPC 775 encoder is used to synchronously measure the rotational speed of the shaft system.

The bearing conditions include normal condition (NC), inner-race fault (IF), outer-race fault (OF), rolling-element fault (RF), and compound fault (CF). The sampling frequency is 200 kHz, and the acquisition duration of each signal is 10 s. To simulate different variable-speed operating conditions, four speed-variation patterns are considered: acceleration, deceleration, acceleration followed by deceleration, and deceleration followed by acceleration. The speed range is 10–30 Hz. This dataset contains 60 records, with 12 recordings for each of the five bearing conditions, and each including a vibration acceleration signal and the corresponding speed signal.

#### 4.1.2. Results Analysis

**Figure 6:**
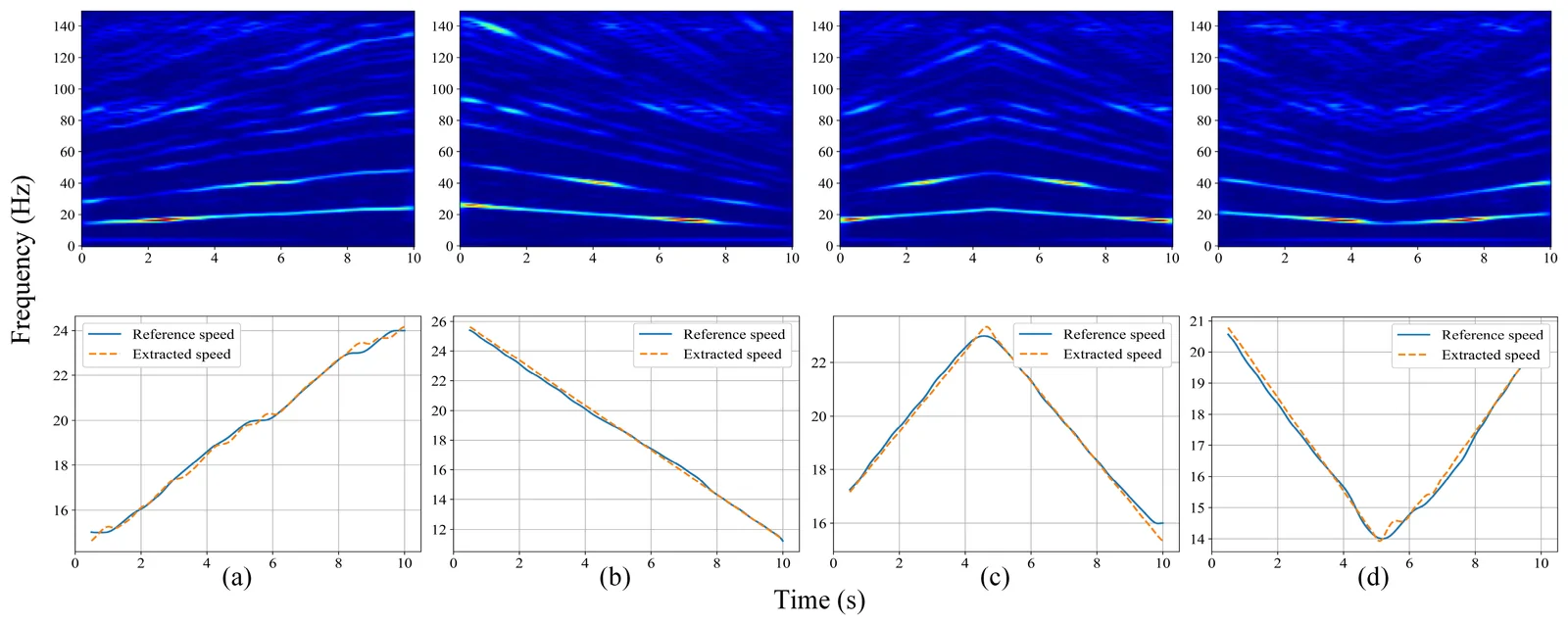
Instantaneous rotational frequency extracted from time–frequency spectra under four speed-variation patterns: (**a**) acceleration; (**b**) deceleration; (**c**) acceleration followed by deceleration; (**d**) deceleration followed by acceleration.

Initially, the raw vibration signal is processed using the tacholess rotational-frequency extraction method proposed in Section 2. After obtaining the time–frequency spectrum through STFT, the initial point of the rotational-frequency ridge is determined by searching the harmonic amplitude summation. Subsequently, the complete rotational-frequency ridge is extracted by combining bilateral-filter-based Canny edge detection with the improved cost-function-based ridge tracking algorithm. Finally, cubic spline interpolation is utilised to fit a continuous and smooth instantaneous rotational-frequency curve. Figure 6 presents the time–frequency spectra and the corresponding instantaneous rotational-frequency extraction results under the four speed-variation patterns. The blue solid curve represents the reference rotational frequency extracted from the key phase signal, while the orange dashed curve represents the instantaneous rotational frequency extracted by the proposed method. The experiment results show that the tacholess rotational-frequency estimation method achieves accurate localisation and comprehensive extraction of the rotational-frequency ridges associated with the signal, even in the presence of time–frequency background interference. The extracted ridge trajectories exhibit pronounced continuity and clarity, confirming robust feature tracking performance.

To further quantitatively evaluate the extraction accuracy of instantaneous rotational frequency, the mean absolute error (MAE), root mean square error (RMSE), and mean absolute percentage error (MAPE) between the extracted rotational frequency and the encoder-measured rotational frequency were calculated for each of the 15 experimental records under each of the four speed-variation patterns. The corresponding metrics were then averaged; the results are presented in Table 2. For all four patterns, the MAE values are below 0.15 Hz, the RMSE values are below 0.18 Hz, and the MAPE values are below 0.85%, indicating that the proposed method can accurately track the instantaneous rotational frequency under different speed-variation patterns. These results demonstrate that the time–frequency ridge extraction method incorporating bilateral-filter-based Canny edge constraints and the improved cost function exhibits favourable adaptability and stability under different forms of speed variation, thereby providing reliable instantaneous rotational-frequency information for the subsequent construction of single-rotation-cycle samples.

The purpose of the rotational-frequency estimation module in this study is to provide sufficiently accurate rotational information for subsequent cycle-synchronous sample construction, rather than to establish a standalone benchmark for tacholess speed-estimation algorithms. Therefore, the independently measured key-phase signal is adopted as the reference for directly evaluating the estimation accuracy.

To construct the cross-condition diagnosis task, cycle-synchronous samples were divided according to their instantaneous rotational frequencies. For each cycle window, its representative rotational frequency was defined as the mean instantaneous rotational frequency over all samples contained in that window. Samples with instantaneous rotational frequencies below 20 Hz were defined as source-condition data, whereas those above and equal to 20 Hz were used exclusively as target-condition test data. The source-condition samples were further divided into training and validation sets at a ratio of 9:1. All four speed-variation patterns were considered in the experiment. The numbers of samples from each bearing condition in the training, validation, and test sets are summarised in Table 3. This explicit partition ensures that model performance is evaluated under a rotational-frequency range that is not included in the training stage. This experimental setting is designed to train the diagnostic model using data from a known rotational-frequency range and to evaluate its fault recognition performance on an unknown rotational-frequency range not involved in the training process. Firstly, the raw vibration signals are downsampled to a sampling frequency of 20 kHz to reduce the computational cost of data processing and model training. Subsequently, the cumulative angular displacement is calculated from the extracted instantaneous rotational frequency, and the raw vibration signal is segmented into single-rotation-cycle samples according to the angular-displacement curve. A sliding-window strategy is adopted for cycle-sample extraction to increase the number of samples and improve the utilisation of features across adjacent cycles. The window length is set to one complete rotation cycle, and the sliding step is set to 0.5 cycles. Considering that the numbers of sampling points contained in different cycles vary under variable-speed conditions, all cycle samples are standardised to a fixed input length of L=1500. For samples containing fewer than 1500 points, the original samples are retained, and zeros are appended to the end of the sequence. For samples containing more than 1500 points, the complete cycle is resampled to 1500 points. Finally, amplitude normalisation is applied to each sample to map its values to the range of [−1, 1]. This operation reduces the influence of amplitude-scale discrepancies across different operating conditions on model training and improves the stability and convergence speed of the training process.

**Figure 7:**
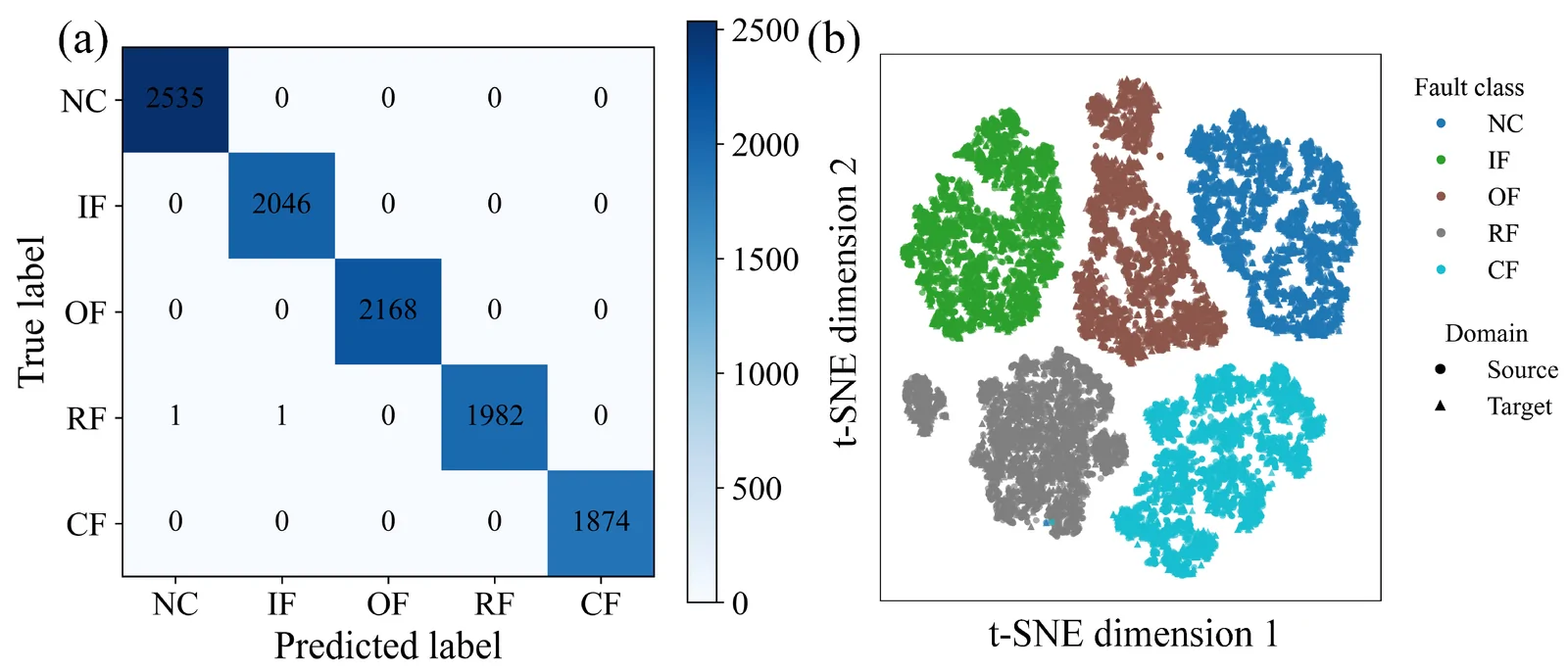
Diagnostic results on the target-condition test dataset: (**a**) confusion matrix of the five bearing health states; (**b**) t-SNE visualisation of the learning latent features.

Following the aforementioned data partitioning and training strategy, the confusion matrix obtained by the model on the target-condition test dataset is shown in Figure 7a. As can be observed, the vast majority of the test samples are correctly identified, with only a small number of rolling-element faults being misclassified. The overall classification accuracy reaches 99.98%. This result indicates that the proposed UDR-CWAE can learn highly discriminative fault features from bearing data acquired under known low-speed operating conditions while maintaining favourable diagnostic performance under unseen high-speed operating conditions that were not included in the training process.

To further investigate the distribution discrepancy between the source and target conditions, the latent features of both domains were jointly projected into a two-dimensional space using t-SNE. Samples are coloured according to fault category, while different markers are used to distinguish the source and target conditions. As shown in Figure 7b, samples belonging to the same fault category from the two rotational-frequency ranges exhibit substantial overlap, whereas samples from different fault categories remain relatively well separated. This result qualitatively suggests that the learned representation reduces operating-condition-related distribution discrepancies while preserving fault-discriminative information. To quantitatively evaluate the distribution discrepancy between the source and target conditions, the MMD was further calculated between their latent representations. This source–target MMD is used only as a post hoc evaluation metric and differs from the MMD regularisation employed during training, which matches the aggregated latent distribution to the predefined prior distribution. For the post hoc evaluation, all latent representations from the source-condition training and validation sets and the target-condition test set were used. The same multi-scale Gaussian RBF kernel used in the latent regularisation, with $\sigma =\left\{1,\;2,\;4,\;8,\;16\right\}$, was adopted. The resulting source–target ${MMD}^{2}$ value was 0.002524, quantitatively characterising the remaining distribution discrepancy between the two rotational-frequency conditions. This observation is consistent with the substantial overlap between source and target samples of the same fault category in the joint t-SNE visualisation.

In addition, five representative deep-learning models, namely CVAE, VAE, CNN, DBN, and SDAE, are selected as comparative baselines to further evaluate the cross-condition fault diagnostic performance of the proposed UDR-CWAE model. To ensure controlled experimental comparison, all models are trained using the same number of epochs, learning rate, and batch size. For the same dataset, all models were retrained 10 times using different random seeds. The mean ± standard deviation of classification accuracy and macro-averaged F1 scores (Macro-F1) are reported in Table 4. Compared with the conventional CNN, UDR-CWAE incorporates a signal reconstruction task and latent-space distribution regularisation in addition to discriminative learning. This design helps reduce the model’s excessive dependence on local statistical characteristics specific to the source operating condition, thereby improving its adaptability to unseen conditions. Compared with VAE and CVAE, the proposed model constrains the discrepancy between the aggregated latent distribution induced by the encoder and the prior distribution, improving the global regularity of the latent space while preserving the representational capacity of the latent variables. Relative to DBN and SDAE, the one-dimensional convolutional architecture adopted by UDR-CWAE is better suited to extracting local impulsive components, periodic waveforms, and multiscale temporal characteristics from vibration signals. Consequently, it exhibits stronger feature extraction and classification capabilities in cross-condition fault diagnosis.

### 4.2. Case Analysis on the SQI Bearing Fault Data

#### 4.2.1. Introduction to SQI Bearing Fault Data

**Figure 8:**
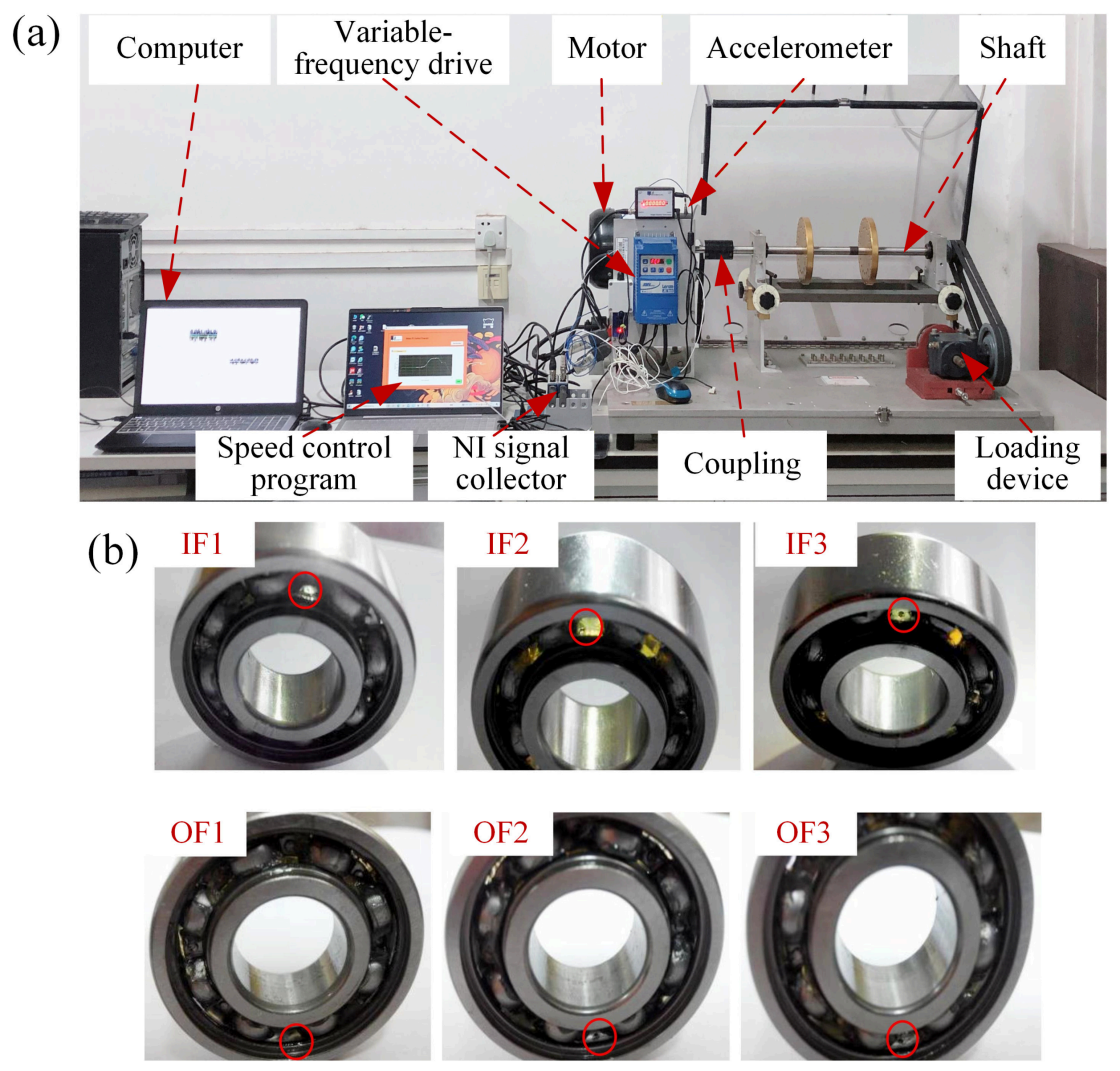
Photographs of the fault simulation test bench and bearing fault specimens: (**a**) SQI test bench and signal acquisition device; (**b**) bearing fault specimens with outer-race spalling, inner-race spalling.

The cross-operating-condition bearing fault experiments were carried out on the SQI bearing fault simulation test bench shown in Figure 8a. The test bench utilises an AC motor as the power input device and employs a Lenze SMVector variable-frequency drive (VFD)(manufactured by Lenze SE, based in Hameln, German) to achieve precise speed control. Torque is transmitted via a flexible coupling to drive the rotor system. During the experimental procedure, the output frequency of the variable-frequency drive is adjusted via computerised control program, causing the drive motor to operate under continuously and dynamically varying variable-frequency speed control conditions. This setup simulates the variable-speed operating characteristics of industrial equipment, thereby providing controllable experimental data to support research on cross-operating-condition fault diagnosis.

The faulty bearing used in the experiment was installed in the drive-end bearing seat inside the motor, and the bearing type was C&U 6203RS. Vibration signals were measured using PCB PIEZOTRONICS 352C68 piezoelectric accelerometers (manufactured by PCB Piezotronics, Inc., based in Depew, NY, USA), which were placed on the motor housing near the drive-end bearing in the vertical and horizontal radial directions. The signals were acquired using an NI9230 data-acquisition card. During each experiment, the commanded VFD frequency varying from 20 to 40 Hz according to a predefined time-varying programme. The programme consisted of alternating quasi-steady operating stages and smooth acceleration and deceleration transitions. The corresponding shaft rotational frequency was measured using the key-phase signal and exhibited a continuous variable-speed profile over the 15 s acquisition period. The same VFD frequency programme was applied to all seven bearing health conditions to ensure consistent operating-condition coverage across different fault categories. The sampling frequency for the vibration signals was set to 25.6 kHz.

A total of seven physical bearing health specimens were considered in the experiment, including the normal condition (NC), three severity levels of inner-race fault (IF), and three severity levels of outer-race fault (OF). For each health condition, 12 independent vibration recordings were acquired. As shown in Figure 8b, defects with different diameters and depths are artificially introduced onto the surfaces of the bearing inner and outer races through machining to represent different levels of fault severity. Specifically, IF1 and OF1 denote slight faults, IF2 and OF2 denote moderate faults, and IF3 and OF3 denote severe faults. The defect dimensions and quantified severity levels of the faulty bearings are listed in Table 5.

#### 4.2.2. Results Analysis

**Figure 9:**
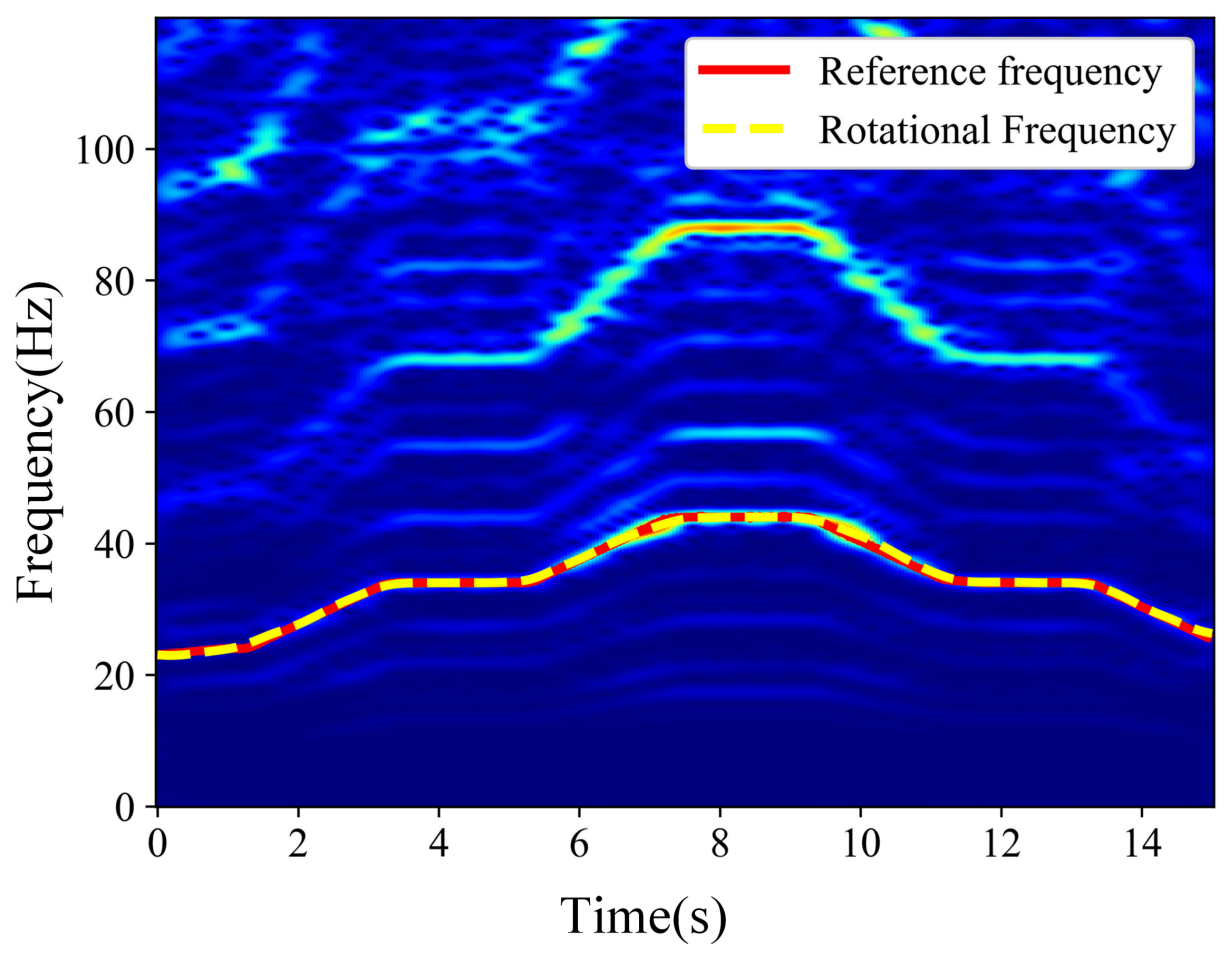
Reference and estimated instantaneous rotational frequencies for the SQI dataset under the predefined variable-speed programme.

Similarly, the raw vibration signal is processed using the tacholess rotational-frequency estimation method proposed in Section 2. Figure 9 presents the time–frequency spectrum and the extracted rotational-frequency curve. The red solid curve represents the reference rotational frequency extracted from the collected key phase signal, while the yellow dashed curve represents the instantaneous rotational frequency extracted by the proposed method. The extracted rotational frequency was compared with the reference obtained from the synchronously acquired key-phase signal. The resulting MAE, RMSE, and MAPE were 0.1325 Hz, 0.1689 Hz, and 0.7122%, respectively, further confirming that the estimated rotational frequency provides sufficiently accurate rotational information for subsequent single-rotation-cycle sample construction.

Following the same window-assignment rule as that used for the Ottawa dataset, the single-rotation-cycle samples with instantaneous rotational frequencies below 30 Hz were assigned to the source condition, whereas those above or equal to 30 Hz were treated as the target condition. The source-condition samples were further divided into training and validation sets at a ratio of 9:1, while the target-condition samples were used exclusively for testing. The resulting per-class sample distributions are provided in Table 6.

To mitigate class imbalance in the source-condition training set, an inverse-frequency class-weighting strategy was applied to the classification loss. For class c, the weight was calculated as

(34) $$w_{c}=\frac{N}{CN_{c}},$$

where N denotes the total number of training samples, C is the number of fault classes, and $N_{c}$ is the number of training samples belonging to class c. The class weights were applied only during model training, whereas the validation and target-condition test sets retained their original class distributions.

**Figure 10:**
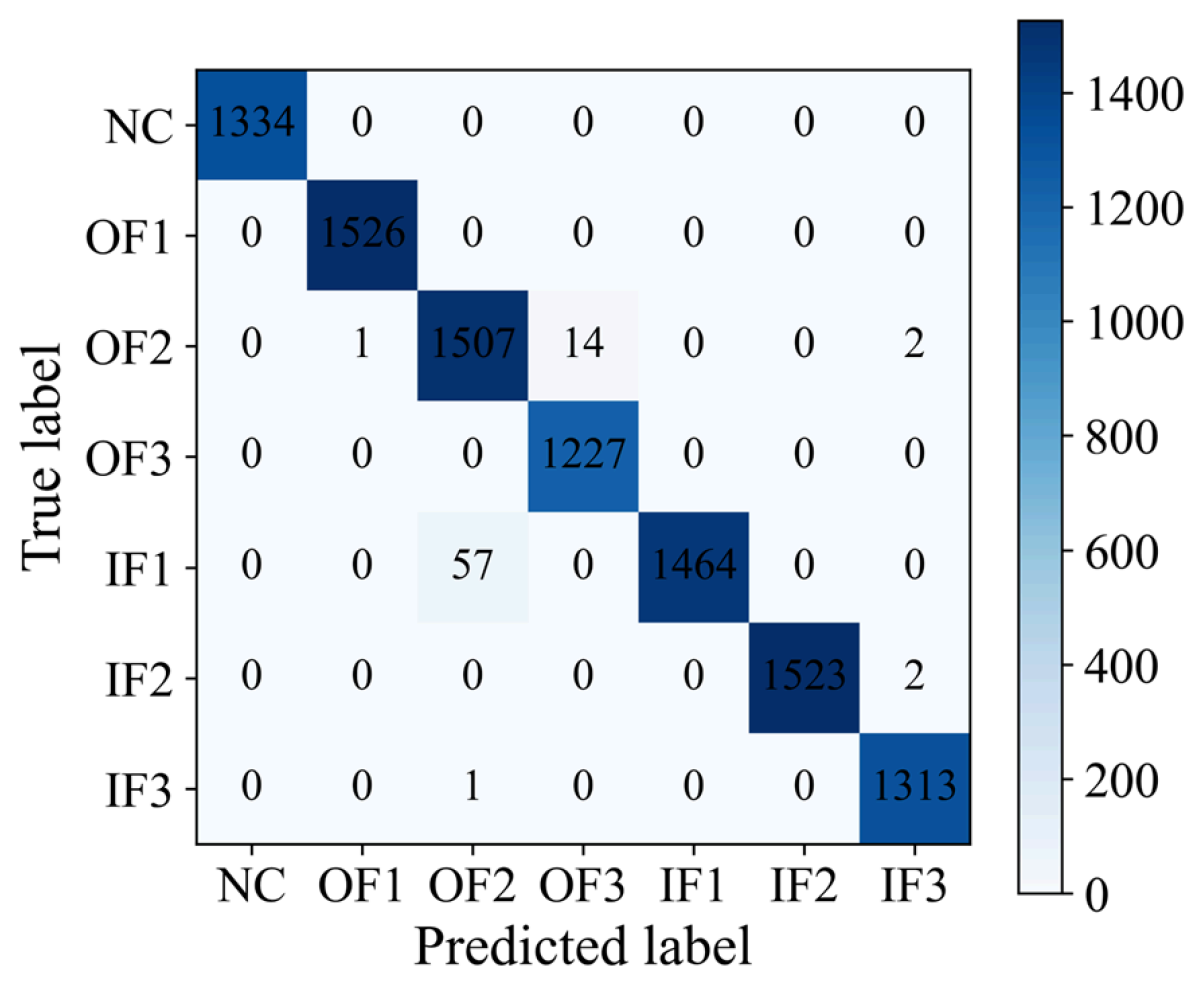
Confusion matrix for the test set of the SQI dataset.

The confusion matrix obtained by UDR-CWAE on the target-condition test dataset is shown in Figure 10. The model achieves an overall classification accuracy of 99.23%. All samples belonging to the NC, OF1, and OF3 classes are correctly classified, while only a very small number of IF2 and IF3 samples are misclassified. These results indicate that the proposed UDR-CWAE can effectively distinguish the normal condition as well as faults with different locations and severity levels. The principal misclassification occurs between IF1 and OF2, with 57 IF1 samples incorrectly classified as OF2. This may be attributed to the relatively weak local impulses generated by slight inner-race faults. During certain operating stages, modulation by the mechanical transmission path may cause their vibration responses to exhibit local waveforms and statistical characteristics similar to those of moderate outer-race faults. In addition, a small number of OF2 samples are misclassified as the adjacent severity levels OF1 or OF3, suggesting that some feature similarity remains between neighbouring fault-severity levels. Overall, the samples in the confusion matrix are highly concentrated along the main diagonal, demonstrating that the proposed model can extract latent features with strong fault discriminability and robustness to operating-condition variations under unseen operating conditions.

### 4.3. Ablation Study

To further investigate the contributions of the latent-space distribution regularisation and uncertainty-guided weighting strategy in UDR-CWAE, ablation experiments are conducted on both the Ottawa and SQI datasets. Three variants of the proposed model are considered: UDR-CWAE without cycle synchronisation, in which the cycle-synchronous sample construction procedure is omitted; UDR-CWAE without MMD, in which the MMD-based latent distribution regularisation term is removed; and UDR-CWAE without uncertainty weighting, in which the uncertainty-guided adaptive weighting strategy is removed while the reconstruction, distribution regularisation, and classification objectives are retained.

For the variant without cycle synchronisation, fixed-length windows L=1500 with a 50% overlap were directly extracted from the time-domain vibration signal. The resulting windows were normalised in the same manner as the cycle-synchronous samples and directly fed into the same UDR-CWAE architecture.

Except for the removed component, the network architecture, data preprocessing procedure, training–validation–test division, and other training settings are kept consistent with those of the complete UDR-CWAE model. The results are presented in Table 7.

As shown in Table 7, removing cycle synchronisation leads to a substantial degradation in diagnostic performance on both datasets. On the Ottawa dataset, the accuracy and Macro-F1 decrease from 99.98% and 99.98% for the complete method to 91.63% and 90.82%, respectively. On the SQI dataset, the corresponding values decrease from 99.23% and 98.79% to 90.77% and 89.52%. These results indicate that constructing cycle-synchronous samples plays an important role in reducing the structural inconsistency of vibration samples caused by variable rotational speed and provides a more consistent representation for subsequent feature learning.

The latent-space distribution regularisation and uncertainty-guided weighting strategy also make clear contributions to the diagnostic performance. On the Ottawa dataset, removing MMD regularisation reduces the accuracy and Macro-F1 to 95.98% and 94.14%, respectively, whereas removing uncertainty weighting results in 95.04% accuracy and 94.36% Macro-F1. A more pronounced degradation is observed on the SQI dataset. Without MMD regularisation, the accuracy and Macro-F1 decrease to 91.78% and 88.30%, respectively, while removing uncertainty weighting reduces them to 90.34% and 87.91%. These results support the contributions of latent prior regularisation and adaptive coordination of the reconstruction, distribution-regularisation, and classification objectives to cross-speed diagnostic performance.

In addition, all three ablated variants exhibit larger standard deviations than the complete method. This tendency is particularly evident when cycle synchronisation is removed, for which the standard deviations of accuracy and Macro-F1 reach 2.30% and 2.76% on the Ottawa dataset and 3.33% and 3.94% on the SQI dataset, respectively. By contrast, the complete method achieves standard deviations of only 0.02–0.03%. This comparison suggests that the combination of cycle-synchronous representation, MMD-based latent distribution regularisation, and uncertainty-guided multi-task weighting not only improves diagnostic accuracy but also provides more stable performance across repeated training runs.

Overall, the complete UDR-CWAE method consistently achieves the highest accuracy and Macro-F1 on both datasets. The ablation results demonstrate that the three investigated components provide complementary contributions: cycle synchronisation reduces speed-induced sample-structure inconsistency at the input-representation level, MMD regularisation promotes a more regularised latent representation, and uncertainty-guided weighting adaptively coordinates the multiple optimisation objectives. Their combination therefore contributes to the improved cross-speed diagnostic performance and stability of the proposed framework.

### 4.4. Discussion

Taken together, the two experiments show that the proposed method achieves high recognition accuracy on two bearing datasets collected under controlled variable-speed conditions, including the publicly available Ottawa dataset and the laboratory SQI test-rig dataset. These results indicate that the proposed method can maintain favourable diagnostic performance under the different variable-speed profiles and fault conditions investigated in the two experimental datasets.

The performance improvement mainly stems from the following three aspects. First, the time–frequency ridge extraction method based on harmonic amplitude summation, edge constraints, and an improved cost function can obtain stable rotational-frequency estimates without an external speed sensor, thereby providing a reliable basis for constructing cycle-synchronised samples. Second, the construction of single-rotation-cycle samples ensures that each input sample corresponds to one complete rotation of the equipment, reducing the sample-structure inconsistency caused by rotational-speed variation. Finally, UDR-CWAE constrains the latent-space structure through distribution regularisation and adaptively coordinates multi-task losses using the uncertainty-weighting strategy, thereby improving the generalisation capability of the model under unknown operating conditions.

Nevertheless, the proposed method still has room for further improvement. First, the present experiments primarily characterise cycle-level generalisation between non-overlapping rotational-frequency ranges. Although cross-subset raw-sample overlap is excluded, the evaluation does not constitute a fully independent cross-record or cross-bearing generalisation scenario. Further validation using entirely independent acquisition runs and bearing specimens will therefore be considered in future work. Second, the time–frequency ridge extraction process still involves several parameters, such as the search frequency band, harmonic order, and edge-detection threshold. More adaptive parameter-selection methods can therefore be investigated in subsequent studies. Third, during the long-term operation of industrial equipment, wear evolution, environmental temperature changes, and gradual operating-condition drift may also occur. Future work can further incorporate online learning or incremental learning mechanisms to improve the long-term deployment capability of the model.

## 5. Conclusions

To address the problems of enhanced vibration-signal non-stationarity, shifted fault-feature distributions, and degraded generalisation capability of diagnostic models for rolling bearings under unknown operating conditions, this study proposes a cross-condition fault-diagnosis method that integrates tacholess time–frequency ridge extraction with an uncertainty-guided distribution-regularised convolutional Wasserstein autoencoder (UDR-CWAE). First, the proposed method extracts the rotational-frequency ridge from the time–frequency spectrum of the vibration signal, thereby enabling instantaneous rotational-frequency estimation without relying on an external speed sensor. Subsequently, single-rotation-cycle samples are constructed based on the estimated rotational frequency, and normalisation is applied to reduce the sample-structure and amplitude-scale discrepancies among different operating conditions. Finally, UDR-CWAE is used to perform latent feature learning and fault classification on the cycle-synchronised samples. Through distribution regularisation and uncertainty-weighted multi-task optimisation, the diagnostic stability and generalisation capability of the model under unknown operating conditions are improved. Experimental results obtained from the Ottawa bearing fault data and laboratory simulations of bearing vibration signals under varying operating conditions indicate that the UDR-CWAE achieves cycle-level classification accuracies of 99.98% and 99.23%, respectively, under the adopted cross-speed evaluation scheme.

In summary, the proposed method improves the cross-condition fault-diagnosis capability of rolling bearings from two perspectives, namely, signal-level physical synchronised representation and deep latent feature learning. Tacholess time–frequency ridge extraction and single-rotation-cycle sample construction alleviate the feature-drift problem caused by variable rotational speed, while UDR-CWAE further improves the regularity and discriminability of latent feature representations under unseen rotational-frequency conditions.

Several limitations remain in the present study. The experimental validation is currently limited to bearing datasets and does not fully cover more complex operating conditions, such as wider load variations, severe noise, and highly irregular speed fluctuations. Future work will therefore focus on validating the proposed method under variable-load conditions and real industrial operating environments, as well as further improving the robustness and real-time applicability of the tacholess diagnostic framework.

## Figures and Tables

**Table 1:** Architecture of the proposed UDR-CWAE.

Module	Configuration
Encoder	Conv1d (1 → 32, k = 9, s = 2) + BN + ReLU + MP (2) + Dropout (0.2); Conv1d (32 → 64, k = 7, s = 2) + BN + ReLU + MP (2) + Dropout (0.2); Conv1d (64 → 128, k = 5, s = 2) + BN + ReLU + AAP (4);
Latent layer	Fatten + FC (→32) + MMD
Decoder	FC (32 → 128) + ReLU; ConvTranspose1d (128 → 64, k = 4, s = 2) + BN + ReLU; ConvTranspose1d (64 → 32, k = 4, s = 2) + BN + ReLU; ConvTranspose1d (32 → 16, k = 4, s = 2) + BN + ReLU; Conv1d (16 → 1) + Tanh;
Classifier	FC (32 → 64) + ReLU + Dropout (0.2) + FC (64 → C)

**Table 2:** Quantitative evaluation of instantaneous rotational frequency extracted under different speed-variation patterns.

Speed-Variation Patterns	MAE (Hz)	RMSE (Hz)	MAPE (%)
Acceleration	0.1171	0.1519	0.6101
Deceleration	0.1489	0.1780	0.7568
Acceleration followed by deceleration	0.1252	0.1638	0.6617
Deceleration followed by acceleration	0.1452	0.1745	0.8486

**Table 3:** Distribution of cycle-synchronous samples for the Ottawa dataset.

Bearing State	Training (<20 Hz)	Validation (<20 Hz)	Test (≥20 Hz)	Total
NC	2017	224	2535	4776
IF	2457	273	2046	4776
OF	2347	261	2168	4776
RF	2513	279	1984	4776
CF	2611	291	1874	4776
Total	11,945	1328	10,607	23,800

**Table 4:** Comparison of cross-condition diagnostic performance among different models on the Ottawa dataset.

Models	Average Accuracy (%)	Average Macro-F1 (%)
UDR-CWAE	99.98 ± 0.02	99.98 ± 0.02
CVAE	95.87 ± 0.35	96.41 ± 0.38
VAE	96.12 ± 0.82	95.81 ± 0.87
CNN	94.33 ± 1.03	94.20 ± 1.10
DBN	75.72 ± 2.79	74.73 ± 2.21
SDAE	33.40 ± 3.47	24.62 ± 4.12

**Table 5:** Fault levels of the test bearing.

Fault Dimensions	Fault Levels of Bearing		
	Slight	Moderate	Severe
S ($mm^{2}$)	4	8	12
Depth (mm)	0.5	1	1.5

**Table 6:** Distribution of cycle-synchronous samples for the SQI dataset.

State	Training (<30 Hz)	Validation (<30 Hz)	Test (≥30 Hz)	Total
NC	1942	216	1334	3492
OF1	1984	221	1526	3731
OF2	1967	218	1524	3709
OF3	1153	128	1227	2508
IF1	1530	170	1521	3221
IF2	1500	167	1525	3192
IF3	1351	150	1314	2815
Total	11,427	1270	9971	22,668

**Table 7:** Ablation results of UDR-CWAE on the Ottawa and SQI datasets.

Model	Ottawa Accuracy (%)	Ottawa Macro-F1 (%)	SQI Accuracy (%)	SQI Macro-F1 (%)
UDR-CWAE without cycle synchronisation	91.63 ± 2.30	90.82 ± 2.76	90.77 ± 3.33	89.52 ± 3.94
UDR-CWAE without MMD	95.98 ± 0.79	94.14 ± 0.93	91.78 ± 1.76	88.30 ± 1.95
UDR-CWAE without uncertainty weighting	95.04 ± 0.99	94.36 ± 1.14	90.34 ± 1.03	87.91 ± 1.21
UDR-CWAE	99.98 ± 0.02	99.98 ± 0.02	99.23 ± 0.03	98.79 ± 0.03

## Data Availability

The datasets analysed during the current study are available from the corresponding authors on reasonable request.

## Abbreviations

The following abbreviations are used in this manuscript:

STFT	Short-time Fourier transform
KL	Kullback–Leibler
WAE	Wasserstein autoencoder
MMD	Maximum mean discrepancy
UDR-CWAE	Uncertainty-guided distribution-regularised convolutional Wasserstein autoencoder
CNN	Convolutional Neural Network
SVM	Support Vector Machine
VAE	Variational Autoencoder
GAN	Generative Adversarial Network
